# The digital code driven autonomous synthesis of ibuprofen automated in a 3D-printer-based robot

**DOI:** 10.3762/bjoc.12.276

**Published:** 2016-12-19

**Authors:** Philip J Kitson, Stefan Glatzel, Leroy Cronin

**Affiliations:** 1 WestCHEM, School of Chemistry, The University of Glasgow, University Avenue, Glasgow G12 8QQ, UK

**Keywords:** 3D printing, digitising chemistry, ibuprofen, laboratory robotics, open source, reaction ware

## Abstract

An automated synthesis robot was constructed by modifying an open source 3D printing platform. The resulting automated system was used to 3D print reaction vessels (reactionware) of differing internal volumes using polypropylene feedstock via a fused deposition modeling 3D printing approach and subsequently make use of these fabricated vessels to synthesize the nonsteroidal anti-inflammatory drug ibuprofen via a consecutive one-pot three-step approach. The synthesis of ibuprofen could be achieved on different scales simply by adjusting the parameters in the robot control software. The software for controlling the synthesis robot was written in the python programming language and hard-coded for the synthesis of ibuprofen by the method described, opening possibilities for the sharing of validated synthetic ‘programs’ which can run on similar low cost, user-constructed robotic platforms towards an ‘open-source’ regime in the area of chemical synthesis.

## Introduction

The rapid expansion of 3D-printing technologies in recent decades has been one of the most promising developments in the fields of science and engineering [1]. This technology, along with the open-source ethos and large, committed user and developer base from which it benefits, has driven innovation in many areas of industrial and technological activity, from distributed manufacturing [2] to practical applications in the areas of medicine [3–4] and biology [5–6]. The use of 3D printers and 3D-printed objects has expanded rapidly, with this technology being applied to scientific disciplines as diverse as biomedical research [7–9], soft robotics [10–11] and materials science [12]. Our group has recently been investigating the use of 3D printing in the chemical sciences, in particular its potential to create ‘reactionware’ [13], that is, chemical reactors where the control which 3D printing offers over the topology, geometry and composition of a reactor [14] can have a significant influence on the reaction outcomes. This utility has so far been demonstrated for a number of applications, from inorganic and organic synthetic [15–16] chemistry to hydrothermal synthesis [17], flow applications [18] and analytical chemistry [19]. One area of research where 3D printers themselves, rather than the products of 3D printing could have a large impact is in the field of laboratory automation.

The automation of laboratory processes has been continuing for as long as the technical abilities and engineering capacities have existed, with the first examples of such equipment appearing in the second half of the nineteenth century [20]. The development of such automation in industrial settings has been rapid, with the inherent flexibility of work in research laboratories leading to much slower adoption of routine automation. One of the barriers to large-scale adoption of laboratory automation technologies has been the traditionally high cost of such equipment which is often optimized for very specific routine tasks [21]. Indeed, one area in which these technologies have been slow to develop has been in the area of synthetic organic chemistry. In this field automation has largely been limited to flow chemistries for specific synthetic pathways [22]. Recently however more versatile equipment and synthetic strategies have been developed to cope with a broader range of target syntheses [23]. Whilst this equipment offers good value for high precision automation of these tasks the expansion of open-source technologies [24] such as 3D printing in the last decade dramatically expands the scope for versatile, low-cost robotics to become a practical reality across a range of modern scientific disciplines [25]. One of the most common types of user-built 3D printers is the RepRap, which has a large online, open-source support community for both hardware development of the printer as well as open-source software development, making it an ideal base for the production of automated laboratory equipment. RepRap 3D printers are often available in kit form and are inexpensive when compared to other laboratory equipment. A basic 3D printer capable of being modified to automate some laboratory functions can cost in the region of 600–700 €.

Herein we present the modification of a RepRap 3D printer to incorporate liquid handling components such that it can act as a unitary chemical synthesis robot which is capable of fabricating (3D printing) a reaction vessel and subsequently performing the complete synthesis of the common drug ibuprofen. Such low-cost, versatile robots could be adapted for use in a variety of settings, from developing laboratories and use in educational institutions to eventually expanding into a distributed manufacturing regime for chemical products.

## Results and Discussion

### RepRap 3D printer modification

**Figure 1:**
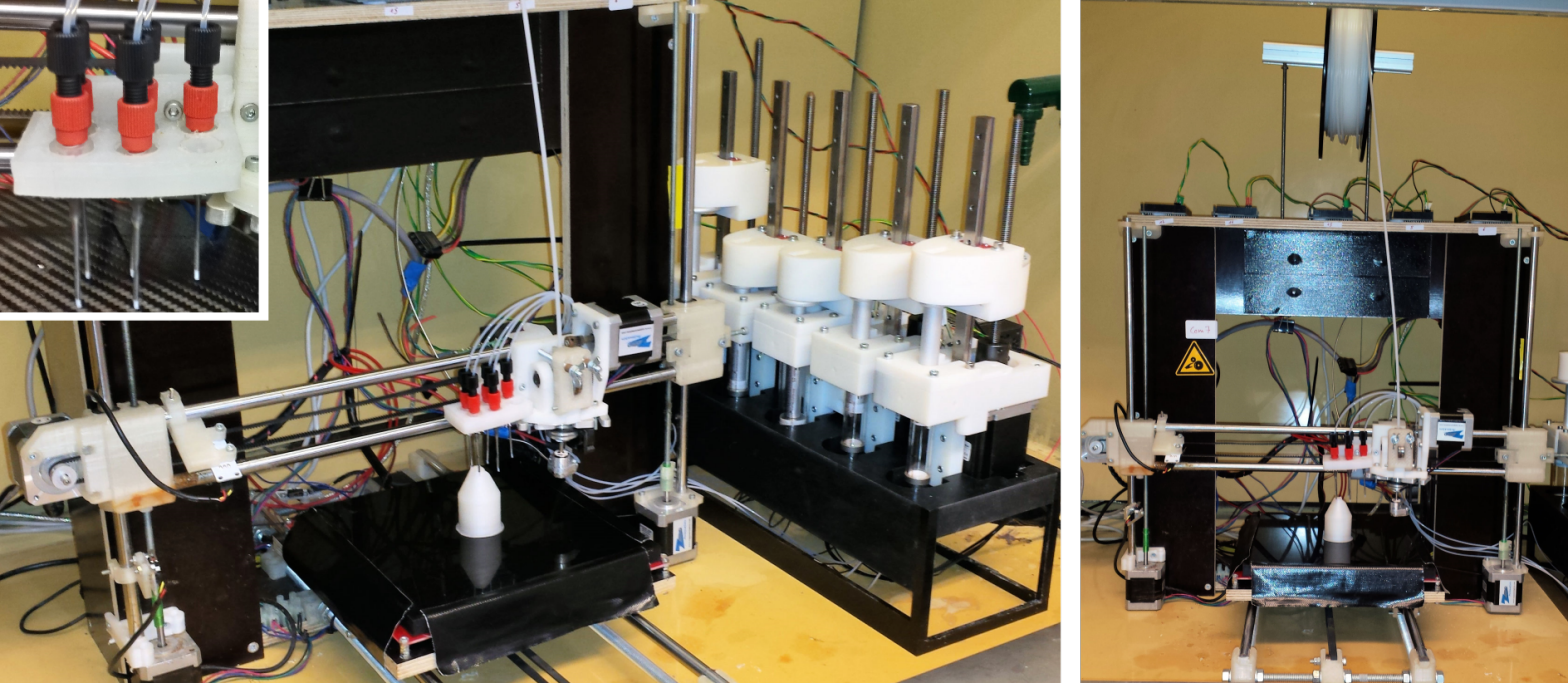
Prusa i3 RepRap printer modified for the automated synthesis of ibuprofen. Left: Full view of robotic platform set-up with a 3D-printed reaction vessel. Left inset: Dispensing needle carriage for 3D printing/liquid deposition. Right: Front view of the 3D-printing section of the robotic set-up with a 3D-printed reaction vessel showing the PP feedstock for reaction-vessel printing.

The RepRap model modified for use was a prusa i3 model (see Figure 1). This is a fused deposition modelling (FDM)-type 3D printer, meaning it works on the principle of using a movable heated print head which extrudes molten or semi-molten material in pre-defined patterns onto a print bed by moving the heated extruder in the *x* and *y* directions. The print head is then incrementally raised in the *z* direction and the printing process repeated to produce the final object. Traditionally parts of this 3D printer are constructed using components which are themselves 3D printed. These components would often be made from polylactic acid (PLA), a widely used 3D printing material. It was found, however, that it was better to construct certain components of the printer from 3D-printed polypropylene (PP) as PLA components degraded quickly if exposed to the chemical environment of a fume hood. The robot was required to have the capacity to both 3D print (for the reaction vessel) and dispense liquids, so the 3D-printing carriage was modified to incorporate both a heated extruder for 3D printing as well as a holder for the polytetrafluoroethylene (PTFE)-lined dispensing needles required for the liquid handling part (see Supporting Information File 1 for further details). These needles were connected by PTFE tubing (internal diameter 0.8 mm) to a number of automatable syringe pumps which have been developed by our group. These pumps were controlled individually by dedicated Arduino control boards and were coordinated via the process-control software developed for the robot. It was determined that the minimum number of pumps necessary to effect the synthesis of ibuprofen by our chosen route was five, to accommodate the starting materials and reagents required.

### Synthetic strategy

**Scheme 1:**
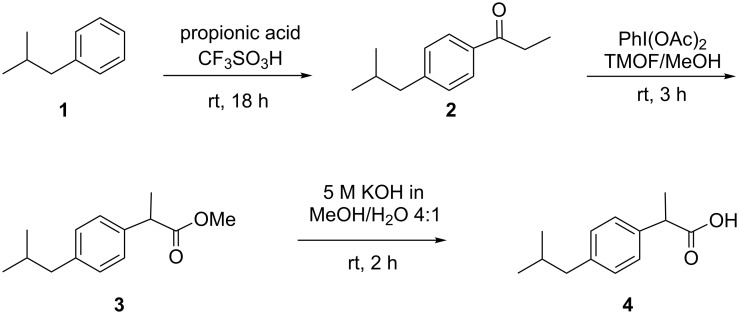
Synthetic route chosen for automated synthesis robot.

The synthesis modified for use with our automated synthetic platform is a three-step synthesis of the popular nonsteroidal anti-inflammatory drug ibuprofen ((*R,S*)-2-(4-(2-methylpropyl)phenyl)propanoic acid) starting from isobutylbenzene and propanoic acid (see Scheme 1). These starting materials undergo a Friedel–Crafts acylation using trifluoromethanesulfonic (triflic) acid (CF_3_SO_3_H) as the Lewis acid catalyst to yield 4-isobutylpropiophenone (**2**). Once this is complete a solution of di(acetoxy)phenyl iodide (PhI(OAc)_2_) and trimethyl orthoformate (TMOF) in methanol (MeOH) is added to the reaction mixture in order to induce a 1,2-aryl migration to produce the ibuprofen methyl ester (**3**). The latter is then hydrolysed in the final step by a potassium hydroxide solution to produce the desired product **4** which can be retrieved after acidic work-up and column chromatography. This synthetic approach was developed by McQuade and co-workers [26] for the puposes of a continuous-flow synthesis of ibuprofen. The reaction was designed specifically such that the byproducts and excess reagents of each step were compatible with the subsequent transformations, eliminating the need for isolation and purification of intermediate products. This approach suited the development of our synthesis robot as the reaction could be performed in a one-pot manner, minimising the liquid handling necessary during the reaction sequence.

**Figure 2:**
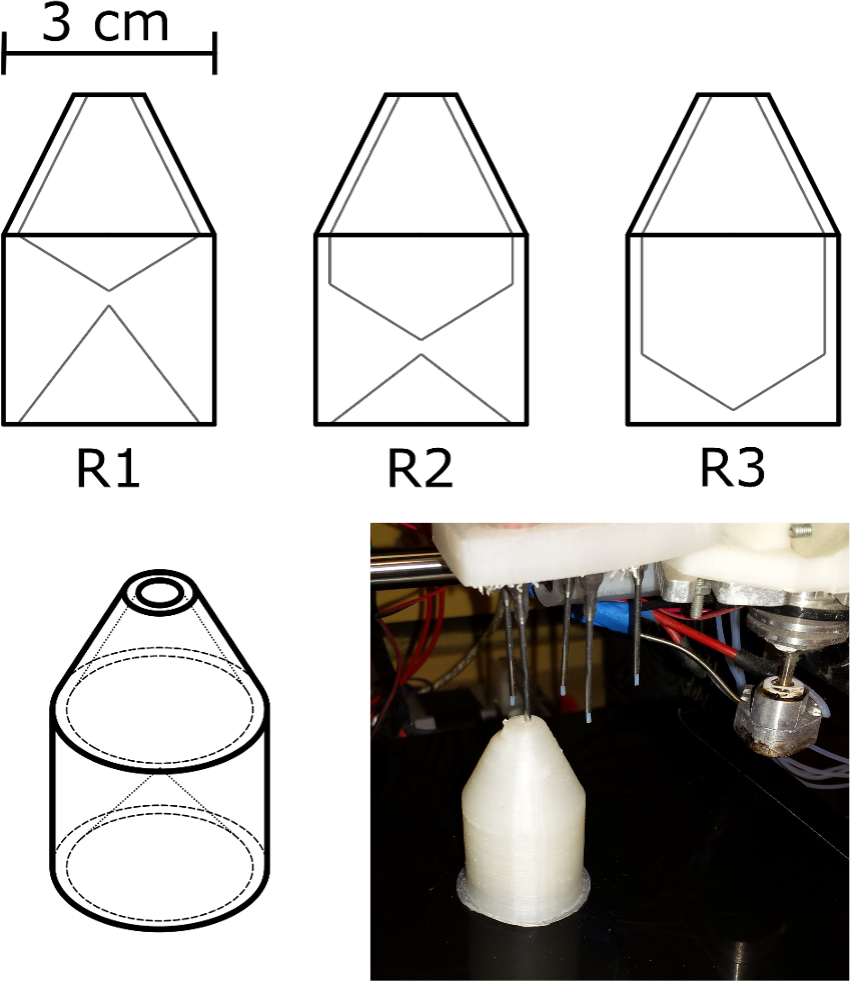
Top: The three reaction vessels printed for ibuprofen synthesis on different scales; bottom left: isometric representation of a reaction vessel; bottom right: reaction vessel in situ during the synthesis of ibuprofen, showing the insertion of the PTFE-lined dispensing needle into the vessel.

The synthetic route was modified to suit the capabilities of the automated robotic platform. For example it was not possible to completely seal the reaction vessel for the duration of the reaction, so it was not feasible to perform the reactions under inert gas atmosphere or to completely exclude atmospheric moisture from the reactions. The reaction vessels were designed to have a small aperture wide enough for only the insertion of the dispensing needle for each chemical to minimise as much as possible the interaction between the reaction and the outside atmosphere (see Figure 2). There were three reaction vessels used for the synthesis, with different capacities depending on the scale of the reaction performed. All vessels were printed using PP, a 3D-printable material which we have found to be compatible with a wide range of chemistries, including those used in this synthesis. The vessels were outwardly similar, but varied in internal volume with capacities with R1, R2 and R3 having total internal volumes of 5.96, 9.68 and 14.99 cm^3^, respectively. In order to effectively print the PP reaction vessels it was necessary to replace the standard carbon fibre or glass-printing bed of the RepRap with a PP plate leading to better adhesion of the PP during printing. The print settings were adjusted such that the reaction vessels could be readily removed after the completion of the synthesis ready to repeat the process (see Supporting Information File 1 for more details).

One of the drawbacks of using a PP-printing bed for the reactor fabrication was that, due to the poor thermal conductivity of PP, we were unable to use the RepRap’s standard heated print bed to effectively heat the reaction mixture. This meant that the reactions of the sequence would have to be carried out at room temperature, leading to longer reaction times to achieve significant conversions for each of the reactions. Similarly, as it was desired that as much as possible of the equipment required for carrying out the reaction be contained entirely within the automated robotic platform it was decided not to include magnetic stirring of the reaction mixture. This would have involved the introduction of a magnetic stirring bar which could not be 3D printed and would have had to be supplied externally. In order to ensure an efficient mixing of the materials, therefore, the *x–y* carriage of the 3D-printing platform was programmed to oscillate rapidly in the *y* axis. The speed and amplitude of this oscillation could be adjusted as parameters in the control software, and were optimised at amplitude of oscillation of 30 mm at a speed of 50 mm s^−1^ for the automated reaction sequence. This proved to be sufficient for the effective mixing of the reaction media on the scales of the reaction vessels printed.

Taking all of these considerations into account the control software for the synthesis robot was designed to coordinate the movements of the 3D printer and liquid handling components in order to achieve the reaction vessel fabrication and chemical processing required for the synthesis of ibuprofen.

### Process control software

**Scheme 2:**
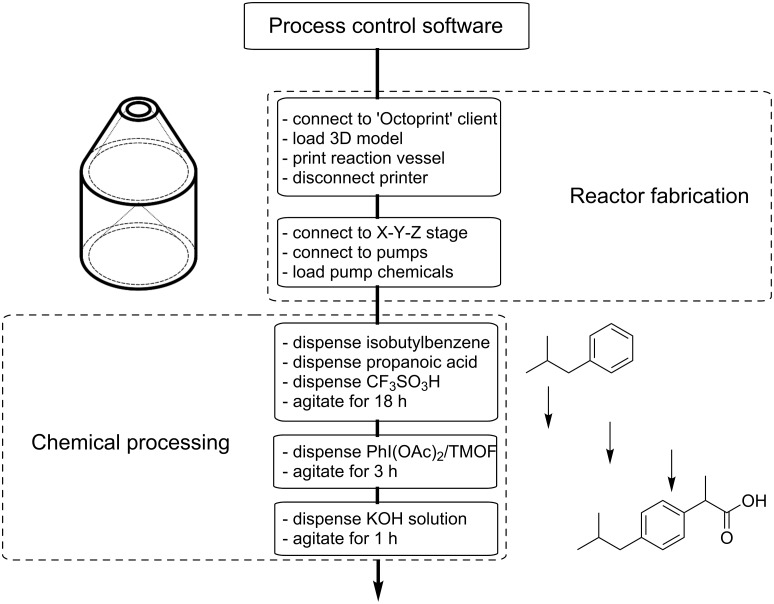
The digitisation of the synthesis of ibuprofen. This flow diagram shows the individual steps of the process control software written to control the chemical synthesis robot.

The software control of the RepRap is also open source allowing the printer to be easily interfaced with user-developed modifications, allowing us to produce our own software for coordinating the 3D printing, liquid handling and reaction timing (see Scheme 2). The software to control our robotic platform was written in Python and the full source code is available from the authors. The control software was designed to be hard-coded for the specific actions required to synthesise Ibuprofen in the three-step synthesis described above, although it would be possible to build on the structure of the software to develop generic modules for liquid handling associated with the modified 3D-printer design, which could then be used to control the robot for a variety of synthetic applications. In this case the focus was on producing a single piece of software which could achieve the fabrication of a reaction vessel, and complete the synthesis of ibuprofen without human intervention other than to ensure the robot was supplied with the necessary reagents and materials (chemical starting materials and thermoplastic stock for the printing of the reaction vessel). This code could then act as a fully self-contained set of synthesis procedures which could be shared with other users as a pre-validated synthesis program to be used with similar robotic systems to achieve the same synthesis.

The control software was designed to first print the reaction vessel used for the ibuprofen synthesis. The software uses the API of an open source 3D-printer control software called OctoPrint (run from source code, available at http://octoprint.org/), which is used to send the gcode (i.e., the 3D-printing instructions) to the printer and perform the 3D printing of the reaction vessel. This process takes about two hours. After this the OctoPrint connection to the printer is terminated and the program connects directly to the firmware of the printer through a serial connection. This connection is used to send the movement commands the printer. The control software has a set definition of the position of each of the dispensing needles for the individual chemicals and the reaction vessel is designed in such a way that the opening for the vessel is positioned at the centre point of the print bed to ease the programming of the dispensing positions. Parameters such as the volume of each chemical to be dispensed into the robot, the amount by which the robot should overdraw each chemical (i.e., the dispensing volume plus an arbitrary amount to ensure that the full volume can be dispensed during the synthesis) are collected together in the source code as user definable variables which can be set depending on the scale of the synthesis required.

For debugging and optimisation purposes all of the programmed routines contain various debug levels where the program intercepts at predefined points (or in the highest level at every step) and waits for the user to acknowledge to go ahead or skip the current step. This is particularly useful when only a small part of the program needs to be tested in the bigger context of the rest of the source code. All working steps can simply be skipped without actually deactivating the responsible code.

### Automated ibuprofen synthesis

The automated synthesis of ibuprofen is initiated with the running of the control software which then proceeds to print the specified reaction vessel. Once this was complete the appropriate pumps were charged with the starting materials solutions (see Table 1) and the control software continued with the automated reaction scheme until the final product solution was ready to be collected. The total time for the completed synthesis was approximately 24 hours, during which time the synthesis robot required little to no interaction from human operators.

**Table 1:** Contents of the automated syringe pumps controlled by the automated synthesis robot.

Pump no.	Contents

1	isobutylbenzene^a^
2	propanoic acid^a^
3	triflic acid^b^
4	PhI(OAc)_2_/MeOH/TMOF^c^
5	KOH^d^

^a^1.05 M in CHCl_3_; ^b^neat; ^c^PhI(OAc)_2_ was prepared as a 1.4 M solution in a mixture of MeOH/TMOF (1:0.8 v/v); ^d^5 M in MeOH/H_2_O 4:1.

For the first reaction, chloroform solutions of isobutylbenzene (1.0 M) and propanoic acid (1.0 M) were deposited into the reaction vessel, followed by the dropwise addition of triflic acid over the course of 10 min to minimise the exotherm produced. Once this process was completed the dispensing needle is raised from the aperture and the reaction is agitated. After 18 h of agitation the needle corresponding to the PhI(OAc)_2_/TMOF solution is lowered into the aperture of the vessel and this solution is once again dispensed dropwise over the course of 10 min. This is followed by further agitation for 3 h, after which the final solution of KOH (5 M in MeOH/H_2_O 4:1 v/v) is added, again dropwise over the course of 10 min, followed by agitation for 1 h. After this step the robot returns to its home position and the reaction mixture can be retrieved yielding, after acidic work-up and column chromatography, ibuprofen in yields of up to 34% over three steps (average of 6 automated runs). The PP reactors showed no evidence of degradation due to the reaction sequence performed, and could be effectively cleaned for reuse as a reaction vessel, all yields, however, were calculated from fully automated runs of the control software including the reaction vessel printing stage. During the testing phase of the robot, the progress of each of the reactions was monitored by using the debug feature of the process control software. For this progression was paused after each stage and aliquots taken from the reaction mixture to be analysed by NMR to ensure the synthesis was proceeding as planned (giving approximate yields by ^1^H NMR of 71% for the initial acylation step and 64% for the subsequent rearrangement, see Supporting Information File 1). Thus using the automation of the robot enables a ‘debugging’ of the chemical processes as well as the control software. However, once the synthetic procedure and parameters were defined the robot was capable of performing the reaction in an autonomous fashion (for a demonstration of the liquid handling steps of the automated reaction sequence, see Supporting Information File 2). Due to the automated nature of the process, the scale of the synthesis could be modified simply by adjusting the parameters in the process control program and ensuring that the reaction vessel design is appropriate for the reaction scale desired. To this end the synthesis of ibuprofen was completed on three different scales by fabricating different reaction vessels (R1–R3) and varying the software parameters controlling the volumes of each of the reaction solutions deposited. These could be easily tuned in the control software and the volumes required for each of these scales are summarised in Table 2. The yields from each of the scales of reaction described were similar (see Supporting Information File 1), however, it was found that further scale-up by increasing the reaction vessel volume (a fourth reaction vessel, R4, was also produced with an internal volume of 28.12 mL, see Supporting Information File 3 for dimensions) lead to reduced yields and longer reaction times. The maximum yield obtained using larger volume reactor vessels was approximately 12% isolated yield of ibuprofen. However the larger vessels also suffered from repeatability problems with less reliability in the yields obtained. These effects are presumably due to a less efficient mixing of the reaction media by agitation in the larger volume of the reaction vessel. This could be remedied by ‘numbering up’ the reaction vessels that the robotic platform prints in the initial stage and adjusting the control software such that several reactions could be run in parallel to increase the yield of ibuprofen.

**Table 2:** Pump contents and reaction volumes.

Pump number	Withdrawn volume (mL)	Deposited volume (mL)	Reaction vessel

1	1.5	0.2	R1
		0.4	R2
		0.8	R3
2	1.5	0.2	R1
		0.4	R2
		0.8	R3
3	3.0	0.35	R1
		0.7	R2
		1.4	R3
4	10.0	1.5	R1
		3.0	R2
		6.0	R3
5	10.0	2.0	R1
		4.0	R2
		8.0	R3

Once the reaction sequence had been completed the final reaction mixture was removed from the reaction vessel by syringe and diluted with water. After acidic work-up the residue was purified by reversed-phase column chromatography on C18 (60% MeCN/H_2_O) to give ibuprofen (**4**) as a white powder. The isolated and averaged yields obtained from six independent automated runs of the system at different scales are given in Table 3 below. There appears to be little depreciation of efficiency of the reaction sequence on the reaction scales with isolated yields varying from 24% to 38%.

**Table 3:** Isolated ibuprofen yields for automated synthesis.

Reaction vessel	Automated run	Ibuprofen yield, mg (%)	Average yield (%)

R1	1	15.9 (36)	32.1
	2	12.8 (29)	
	3	17.0 (39)	
	4	10.2 (24)	
	5	15.3 (35)	
	6	13.1 (30)	
R2	1	28.5 (33)	34.2
	2	26.7 (31)	
	3	33.2 (38)	
	4	31.0 (36)	
	5	27.5 (32)	
	6	29.9 (35)	
R3	1	60.1 (34)	33.7
	2	57.0 (33)	
	3	61.6 (36)	
	4	58.2 (34)	
	5	60.5 (35)	
	6	51.7 (30)	

## Conclusion

By modifying a relatively inexpensive 3D-printing platform we were able to construct a unitary ‘synthesis robot’ which is capable of autonomously fabricating a reaction vessel and performing the liquid handling steps necessary to effect the synthesis of the common painkiller ibuprofen. This example demonstrates the unique versatility of the current generation of open-source consumer robotic equipment to be modified for use in laboratory automation. Using this synthesis robot we were able to synthesise the popular drug ibuprofen on three different reaction scales using a piece of custom software to control the parameters of the synthesis. Future developments in this field could include the development of further open source solutions to allow robotic platforms to perform more of the routine functions of chemical synthesis such as work-up and purification routines. The widespread use of such low-cost automation of chemical synthesis could allow the development of an ‘open source’ approach to chemical synthesis itself where synthetic routines can be downloaded and tested by any laboratory with the necessary robotic platform, advances in the chemical automation equipment could then run in parallel with advances in the synthetic strategies used. Finally, this work shows how chemical synthesis can be fully digitized into a standalone code and autonomously run on a robotic system. Not only could this potentially overcoming reproducibility issues that can limit the exchange of synthetic chemistry, but allow users to share their code thereby allowing more complex molecules to be designed and made within autonomous chemical robots.

## Supporting Information

Supporting information is available containing full experimental details, the source code of the process control software, along with information on the 3D printing settings for the reactor vessel fabrication. Also available are a video demonstrating the liquid handling for the automated reaction sequence and the .STL digital model files of the reactor vessels fabricated by the robotic platform.

File 1 Full experimental details, the source code of the process control software, along with information on the 3D printing settings for the reactor vessel fabrication.

File 2 Digital 3D model files archive for the reaction vessels used.

File 3 Demonstration video of the liquid handling of the automated reaction sequence.
