# Supplementary material for: The digital code driven autonomous synthesis of ibuprofen automated in a 3D-printer-based robot
Source: Beilstein J Org Chem. 2016 Dec 19;12:2776–83. doi: 10.3762/bjoc.12.276 (PMC5238580; doi:10.3762/bjoc.12.276)
Supplement: File 1 — Full experimental details, the source code of the process control software, along with information on the 3D printing settings for the reactor vessel fabrication. [file Beilstein_J_Org_Chem-12-2776-s001.pdf]

Supporting Information File 1

for

The digital code driven autonomous synthesis of ibuprofen automated in a 3D-printer-based robot

Philip J. Kitson, Stefan Glatzel, and Leroy Cronin*

Address: WestCHEM, School of Chemistry, The University of Glasgow, University Avenue, Glasgow G12 8QQ, UK

Email: Leroy Cronin - Lee.Cronin@glasgow.ac.uk

*Corresponding author

Full experimental details, the source code of the process control software, along with information on the 3D printing settings for the reactor vessel fabrication

Contents

General experimental remarks S3

Robot design and construction S4

3D Printer modifications S4

Process control software S6

Full process control software code. S7

Design software S27

Device model S27

Ibuprofen synthesis S30

General experimental remarks

All chemical reagents and solvents were purchased from Sigma Aldrich and used without further purification.

^{1}H NMR and ^{13}C NMR: ^{1}H NMR and crude ^{13}C NMR spectra were recorded on a Bruker Avance 400 MHz machine at 298 K, and chemical shifts are reported in ppm relative to residual solvent signal (multiplicities are given as s: singlet, d: doublet, t: triplet, q: quartet, m: multiplet, with coupling constants reported in Hz). Final product ^{13}C NMR and two dimensional NMR spectra were recorded on a Bruker Avance III 500 MHz machine at 298 K.

Mass spectrometry: Mass spectra were obtained using a Q-trap, time-of-flight MS (MicroTOF-Q MS) instrument equipped with an electrospray (ESI) source supplied by Bruker Daltonics Ltd. Analysis was carried out in MeOH, collected in positive ion mode. The spectrometer was calibrated with the standard tune-mix to give a precision of ca.1.5 ppm in the region of *m/z* 100–3000.

Robot design and construction

3D Printer modifications

A RepRap 3D printer kit, model Prusa i3, was purchased from RepRap Source (Germany, www.reprapsource.com) and assembled according to manufacturer specifications. The firmware running on the mainboard of the printer is Marlin (https://github.com/MarlinFirmware/Marlin). This printer was then modified to incorporate the required liquid handling elements for the synthesis of ibuprofen.

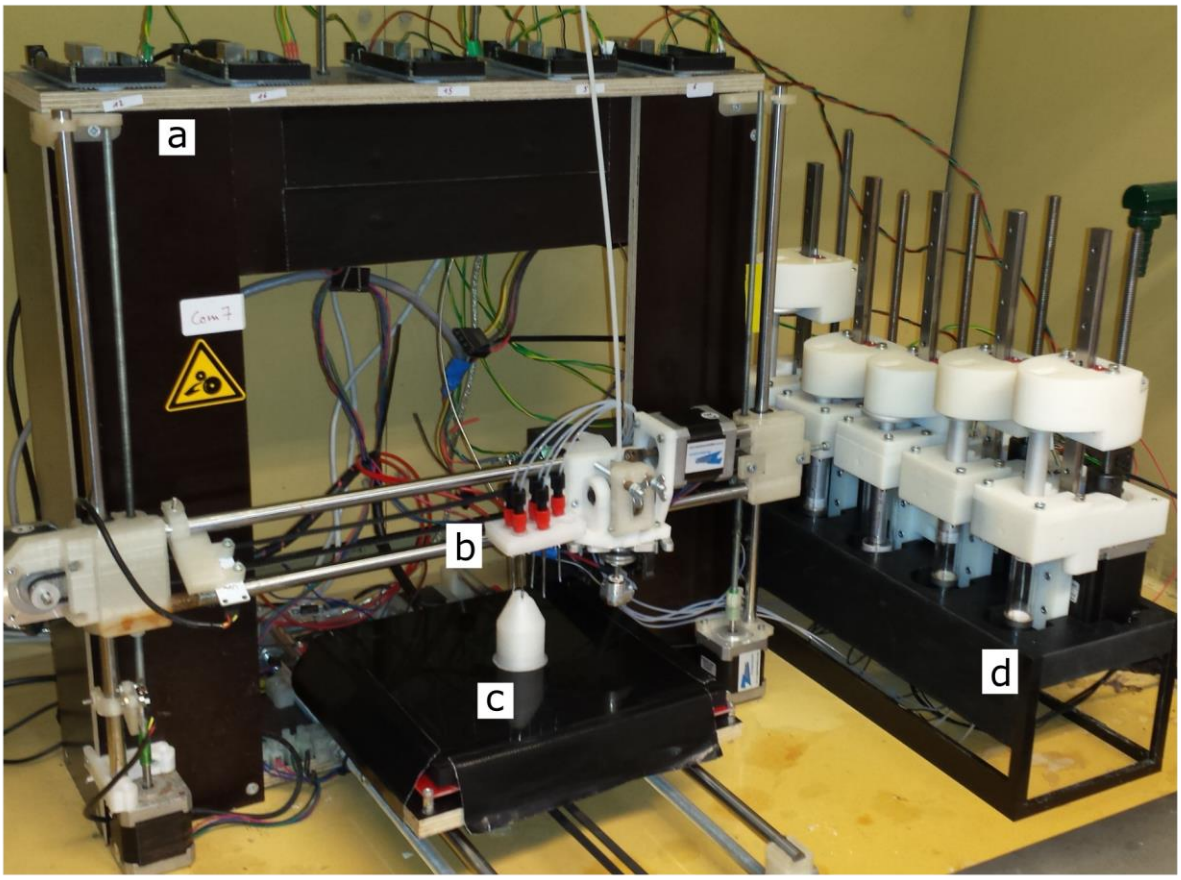

Figure S1: *Prusa i3 RepRap printer modified for the automated synthesis of Ibuprofen. (a) Arduino control boards for in-house developed pumps. (b) Extruder / needle holder carriage for 3Dprinting / liquid deposition. (c) 3D printed reaction vessel. (d) In-house developed pumps for liquid handling.*

We designed a custom holder for the mounting point of the syringes required for the liquid handling capabilities of the robot (Figure S1). This carriage was designed around the existing RepRap extruder mounting points and Luer (Male) to 1/4"-28 Flat Bottom (Female), ETFE/polypropylene connectors from Kinesis (UK, Part #: P-675). This carriage contained space for six Luer connectors, five of which were fitted with PTFE-lined dispensing tips (length: 1.5”; internal diameter: 0.006”; Fisnar Europe Ltd.). These were then connected to PTFE 0.8 mm ID

tubing (kinesis using standard ¼”-28 connectors. This tubing was then connected to in-house developed syringe pumps for liquid handling.

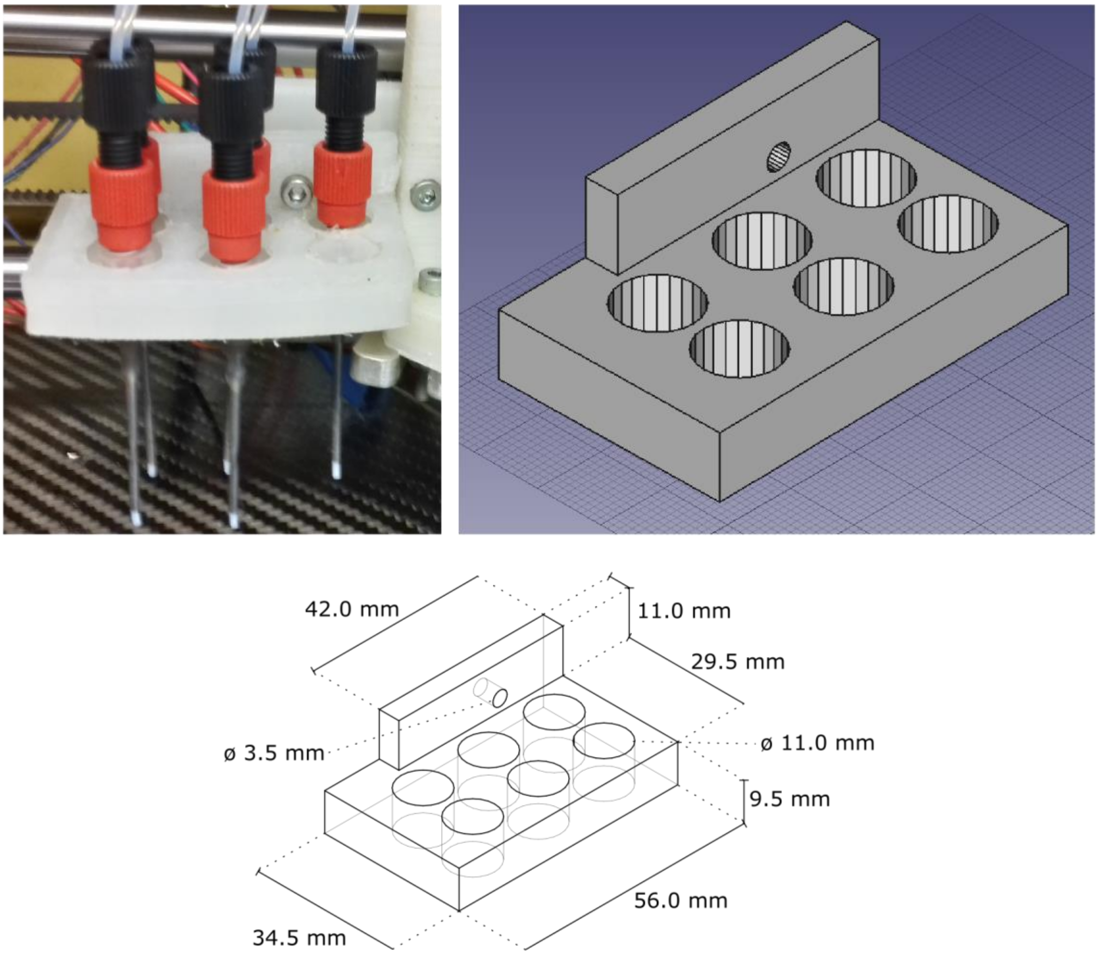

Figure S2:*Top left: Photograph of the 3D printed needle holder carriage in place on the modified 3D printer chassis. Top right:render of the 3D printed needle holder carriage. Bottom: Annotated CAD design of the 3D printed needle holder carriage.*

The designed carriage was exported as an STL (stereo-lithography) file and converted to 3D printer instructions (gcode) using the open-source software slic3r (http://slic3r.org/). The thus obtained carriage file was printed on the same 3D printer using polypropylene (PP) supplied by Barnes plastic welding ltd. Finally, the designed holder was fixed to the exiting X-axis carriage of the 3D printer with two 25 mm M3 screws.

Process control software

The software to control our robotic platform was written in python and developed and run in the Integrated Development Environment PyCharm Community Edition (https://www.jetbrains.com/pycharm/) the 3D printing is handled by the open-source 3D printer web interface Octoprint (http://octoprint.org/) (see Figure S3). The full source code is reproduced below.

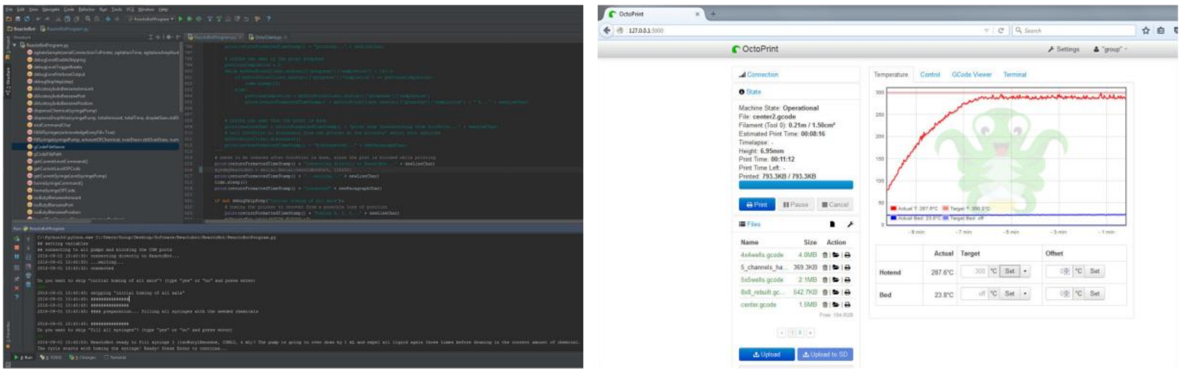

***Figure S3**: L<u>eft</u>: Screenshot of the Process control software running in the PyCharm environment. Right: Sreenshot of the Octoprint 3D printing web interface.*

Full process control software code.

```
# !/usr/bin/env python
#/* ============================================================================
*/
#/*
*/
#/*   ReactoBotIbuprofenSynthesis.py
*/
#/*   (c) 2013 - 2014 Stefan Glatzel, The Cronin Group, University of Glasgow
*/
#/*
*/
#/*   WARNING! This file intentionally has almost no error handling!
*/
#/*   It is solely intended to be a hardcoded synthesis of Ibuprofen.
*/
#/*   It assumes that the robot is in full working order and all the syringes
*/
#/*   are preloaded with the correct chemicals in the correct amounts.
*/
#/*
*/
#/* ============================================================================
*/

# system imports
import datetime
import math
import os
import time

# additional modules
import serial
from OctoClient import OctoClient

print("## setting variables")
#general variables
# various debug levels, level above 2 will trigger every break, level above 5
will produce verbose output from the pumps
debugLevelVerboseOutput =                9
debugLevelTriggerBreaks =                3
debugLevelEnableSkipping =               6
runInDebugLevel =                        10
newLineChar =                            ""
newParagraphChar =                       "\n"
syringePumpLevelReturnString =           "Current syringe level: "
numberOfErrors =                         0
```

```
# general "chemistry" settings
stdOverDraw =                           1
stdNumberOfFillEmptyCycles =            0
stdEquilibrationTime =                  10
stdDropletSize =                        0.1

# necessary command codes for the pumps
getCurrentLevelOPCode =                 10
setAbsoluteOPCode =                     11
setRelativeOPCode =                     12
homeSyringeOPCode =                     19
endCommandChar =                        "\n"

# COM ports number ist one less than in "COM XX" (COM15 -> 14)
isoButylBenzenePort =                   11  # COM12
propanoicAcidPort =                     15  # COM16
triFluoroMethaneSulfonicAcidPort =      14  # COM15
diAcetoxyIodoBenzenePort =              4   # COM5
workUpPort =                            5   # COM6
reactoBotPort =                         6   # COM7

# syringe positions [X, Y, Z]
isoButylBenzenePosition =               [136, 80, 15]
propanoicAcidPosition =                 [170, 80, 15]
triFluoroMethaneSulfonicAcidPosition =  [170, 97, 15]
diAcetoxyIodoBenzenePosition =          [153, 97, 15]
workUpPosition =                        [153, 80, 15]

sufficientZHeight =                     45

# OctoPrint address
octoPrintAddress =                      ""
octoPrintPort =                         ""
octoPrintAPIKey =                       ""

# amounts of all chemicals needed in mL

isoButylBenzeneAmount =                 ##
propanoicAcidAmount =                   ##
triFluoroMethaneSulfonicAcidAmount =    ##
diAcetoxyIodoBenzeneAmount =            ##
workUpAmount =                          ##

# gCode file path for the reaction vessel
gCodeFileName =                         "15mL Reaction Vessel_T2.gcode"
gCodeFilePath =                         os.path.join(os.path.dirname(__file__),
gCodeFileName)

print("## connecting to all pumps and blocking the COM ports")
# establishing the serial connections
standardConnectionSpeed =               9600
isoButylBenzenePump =                   serial.Serial(isoButylBenzenePort,
standardConnectionSpeed)
propanoicAcidPump =                     serial.Serial(propanoicAcidPort,
standardConnectionSpeed)
```

```
triFluoroMethaneSulfonicAcidPump =
serial.Serial(triFluoroMethaneSulfonicAcidPort, standardConnectionSpeed)
diAcetoxyIodoBenzenePump =              serial.Serial(diAcetoxyIodoBenzenePort,
standardConnectionSpeed)
workUpPump =                            serial.Serial(workUpPort,
standardConnectionSpeed)


def returnFormattedTimeStamp():
    """
    returns a formatted time stamp with colon and space to use in print or log
output

    :return: formatted time stamp
    """
    return datetime.datetime.fromtimestamp(time.time()).strftime('%Y-%m-%d
%H:%M:%S') + ": "


def homeSyringeCommand():
    """
    returns the formatted and encoded command string to home a syringe pump

    :return: formatted and encoded command string
    """
    return bytes(str(homeSyringeOPCode) + endCommandChar, encoding="UTF-8")


def getCurrentLevelCommand():
    """
    returns the formatted and encoded command string to get the current level of
a syringe pump

    :return: formatted and encoded command string
    """
    return bytes(str(getCurrentLevelOPCode) + endCommandChar, encoding="UTF-8")


def setRelativeCommand(mLToMove):
    """
    returns the formatted and encoded command string to trigger a relative level
change in a syringe pump

    :param mLToMove: (float) level to go to
    :return: formatted and encoded command string
    """
    return bytes(str(setRelativeOPCode) + " " + str(mLToMove) + endCommandChar,
encoding="UTF-8")


def setAbsoluteCommand(mLToMove):
    """
    returns the formatted and encoded command string to move a syringe pump to
an absolute level
```

```
    :param mLToMove: (float) level to go to
    :return: formatted and encoded command string
    """
    return bytes(str(setAbsoluteOPCode) + " " + str(mLToMove) + endCommandChar,
encoding="UTF-8")

def printErrorMessage(errorMessage, breakAfter=False):
    """
    encases an error message in exclamation marks and breaks after the error if
so desired

    :param errorMessage: (string) message to present
    :param breakAfter: (boolean) wait for acknowledgement by the user (default:
False)
    :return: True
    """
    print("!!!!!!!!!!!!!!!!!!!!!!!!!!!!!!!!!!!!!!!!!")
    print("!!!!!!!!!!!!!!!!!!!!!!!!!!!!!!!!!!!!!!!!!")
    print(returnFormattedTimeStamp() + errorMessage + newLineChar)
    print("!!!!!!!!!!!!!!!!!!!!!!!!!!!!!!!!!!!!!!!!!")
    print("!!!!!!!!!!!!!!!!!!!!!!!!!!!!!!!!!!!!!!!!!")

    if breakAfter or runInDebugLevel > debugLevelTriggerBreaks:
        input("Acknowledge error and continue? Press Enter to continue...")

    return True


def debugSkipStep(step):
    """
    handles debug mode step skipping and returns true or false depending on
whether the user wants to skip a given
    step or not

    :param step: (string) description of the step that the user is queried to
skip
    :return: (boolean) skip step or not
    """
    skipStep = ""
    if runInDebugLevel > debugLevelEnableSkipping:

        while skipStep != "yes":
            skipStep = input("Do you want to skip \"" + step + "\"? (type
\"yes\" or \"no\" and press enter)\n")

            if skipStep == "no":
                break

        if skipStep == "yes":
            print(returnFormattedTimeStamp() + "skipping \"" + step + "\"" +
newLineChar)
            return True
        else:
            return False
    else:
```

```
        return False


def getCurrentSyringeLevel(syringePump):
    # getting the current syringe level
    print(returnFormattedTimeStamp() + "getting current syringe level..." +
newLineChar)

    syringePump.write(getCurrentLevelCommand())

    answer = b""
    while answer[:len(syringePumpLevelReturnString)] !=
bytes(syringePumpLevelReturnString, encoding="UTF-8"):
        answer = syringePump.readline().strip()
        if runInDebugLevel > debugLevelVerboseOutput:
            print("a: " + bytes.decode(answer, encoding="UTF-8"))

    # removing the initial part of the return string
    currentLevel = bytes.decode(answer[len(syringePumpLevelReturnString):],
encoding="UTF-8")
    # splitting the remaining string by spaces
    currentLevel = currentLevel.split()
    # assuming that the first part of that string is the remaining volume in mL
(as float)
    currentLevel = float(currentLevel[0])

    print(returnFormattedTimeStamp() + "current syringe level: " +
str(currentLevel) + " mL" + newLineChar)

    return currentLevel


def dispenseDropWise(syringePump, totalAmount, totalTime,
dropletSize=stdDropletSize):
    """
    method to emulate drop wise dispensing of a liquid over a given period of
time

    :param syringePump: (Serial.serial) pump to communicate with
    :param totalAmount: (float) total amount to dispense in mL
    :param totalTime: (integer) time in minutes during which to dispense the
liquid
    :param dropletSize: (float) dispensing step size in mL (default: 0.1)
    :return: True
    """
    # getting the current syringe level
    currentLevel = getCurrentSyringeLevel(syringePump)

    if currentLevel >= totalAmount:
        print(returnFormattedTimeStamp() + "dispensing " + str(totalAmount) + "
mL in " + str(
            totalTime) + " min." + newLineChar)
        # getting the floor division i.e. 1.45 mL in 0.5 mL droplets will give 2
        totalNumberOfDroplets = totalAmount // dropletSize
        if runInDebugLevel > debugLevelVerboseOutput:
```

```
            print(returnFormattedTimeStamp() + "total number of droplets: " +
str(totalNumberOfDroplets) + newLineChar)
        remainingLiquid = totalAmount - (totalNumberOfDroplets * dropletSize)
        if runInDebugLevel > debugLevelVerboseOutput:
            print(returnFormattedTimeStamp() + "size of last droplet: " +
str(remainingLiquid) + newLineChar)
        timeBetweenDroplets = (totalTime * 60) / totalNumberOfDroplets
        if runInDebugLevel > debugLevelVerboseOutput:
            print(returnFormattedTimeStamp() + "time between droplets: " +
str(timeBetweenDroplets) + newLineChar)
        dispensedDroplets = 0
        while dispensedDroplets < totalNumberOfDroplets:
            dispensedDroplets += 1
            print(returnFormattedTimeStamp() + "dispensing droplet " +
str(dispensedDroplets) +
                  " of " + str(totalNumberOfDroplets) + "(" + str(dropletSize) +
" mL)" + newLineChar)
            syringePump.write(setRelativeCommand(-dropletSize))
            waitForMovementToFinish(syringePump)
            print(returnFormattedTimeStamp() + "waiting " + str(
                timeBetweenDroplets) + " s to next droplet." + newLineChar)
            time.sleep(timeBetweenDroplets)

        if remainingLiquid > 0:
            print(returnFormattedTimeStamp() + "dispensing the remaining liquid
(" + str(
                remainingLiquid) + " mL)" + newLineChar)
            syringePump.write(setRelativeCommand(-remainingLiquid))
            waitForMovementToFinish(syringePump)
    else:
        printErrorMessage("ERROR! Syringe does not contain enough liquid!
(Current level: " + str(currentLevel) +
                          " mL. Requested amount: " + str(totalAmount) + " mL.")

    return True


def waitForMovementToFinish(serialConnectionForListening, waitForM400=False):
    """
    waits on a serial connection to receive "ok" as acknowledgement of a
finished movement.
    if waitForM400 is set it sends "M400" after it receives an initial "ok",
because the Marlin firmware on RepRap
    printers acknowledges received commands directly with "ok" and only signals
finished movements after being "asked"
    to do so with "M400"

    :param serialConnectionForListening: (Serial.serial) serial connection to
listen on
    :param waitForM400: (boolean) the Marlin firmware on RepRap printers
acknowledges received commands with "ok"
            -> send M400 (wait for movement to finish) and wait again for "ok"
for movement finished
    :return: True
    """
```

```
    answer = b""
    while answer != b"ok":
        answer = serialConnectionForListening.readline().strip()
        if runInDebugLevel > debugLevelVerboseOutput:
            print(returnFormattedTimeStamp() + "a: " + bytes.decode(answer, 
encoding="UTF-8") + newLineChar)
    answer = b""

    if waitForM400:
        serialConnectionForListening.write(b"M400\n")
        while answer != b"ok":
            answer = serialConnectionForListening.readline().strip()
            if runInDebugLevel > debugLevelVerboseOutput:
                print(returnFormattedTimeStamp() + "a: " + bytes.decode(answer, 
encoding="UTF-8") + newLineChar)

    return True


def agitateSample(serialConnectionToPrinter, agitationTime, 
agitationAmplitude=10, agitationSpeed=10000):
    """

    :param serialConnectionToPrinter:
    :param agitationTime:
    :param agitationAmplitude:
    :param agitationSpeed:
    :return:
    """
    print(returnFormattedTimeStamp() + "agitating sample for " + 
str(agitationTime / 3600) + " h..." + newLineChar)

    # switch to relative movements
    serialConnectionToPrinter.write(b"G91\n")
    #read the "ok" from the printer, otherwise it will stay in the buffer and 
mess up the next check
    waitForMovementToFinish(serialConnectionToPrinter)

    # set the percentage to 0 and record the start time
    percentDone = 0
    start = time.time()

    while time.time() < start + agitationTime:
        serialConnectionToPrinter.write(
            bytes("G1 Y" + str(agitationAmplitude) + " F" + str(agitationSpeed) 
+ "\n", encoding="UTF-8")
        )
        # wait for movement to finish
        waitForMovementToFinish(serialConnectionToPrinter, True)

        serialConnectionToPrinter.write(
            bytes("G1 Y-" + str(agitationAmplitude) + " F" + str(agitationSpeed) 
+ "\n", encoding="UTF-8")
        )
        # wait for movement to finish
```

```
        waitForMovementToFinish(serialConnectionToPrinter, True)

        if math.floor(100 * (time.time() - start) / agitationTime) >
percentDone:
            percentDone = math.floor(100 * (time.time() - start) /
agitationTime)
            print(
                "{0}: Agitation to {1}% done. Approximately {2} h, {3} min, {4}
sec remaining.{5}".format(
                    returnFormattedTimeStamp(),
                    percentDone,
                    str(math.floor((start + agitationTime - time.time()) /
3600)),
                    str(math.floor((start + agitationTime - time.time() -
(math.floor((start + agitationTime - time.time()) / 3600) * 3600)) / 60)),
                    str(
                        round(
                            start + agitationTime - time.time() - (
                                (math.floor((start + agitationTime - time.time()
- (math.floor((start + agitationTime - time.time()) / 3600) * 3600)) / 60)  *
60) +
                                (math.floor((start + agitationTime -
time.time()) / 3600) * 3600)
                            )
                        )
                    ),
                    newLineChar
                )
            )

    # switch back to absolute movements
    serialConnectionToPrinter.write(b"G90\n")
    #read the "ok" from the printer, otherwise it will stay in the buffer and
mess up the next check
    waitForMovementToFinish(serialConnectionToPrinter)

    return True


def raiseZForXYMovement():
    """


    :return:
    """
    print(returnFormattedTimeStamp() + "raising Z" + newLineChar)
    myReactoBot.write(bytes("G1 Z" + str(sufficientZHeight) + " F100\n",
encoding="UTF-8"))
    # wait for movement to finish
    print(returnFormattedTimeStamp() + "...waiting..." + newLineChar)
    waitForMovementToFinish(myReactoBot, True)
    print(returnFormattedTimeStamp() + "done..." + newParagraphChar)

    return True
```

```
def lowerZForChemicalDispensing(syringePosition):
    """

    :param syringePosition:
    :return:
    """
    print(returnFormattedTimeStamp() + "lowering Z" + newLineChar)
    myReactoBot.write(bytes("G1 Z" + str(syringePosition[2]) + " F100\n",
encoding="UTF-8"))
    # wait for movement to finish
    print(returnFormattedTimeStamp() + "...waiting..." + newLineChar)
    waitForMovementToFinish(myReactoBot, True)
    print(returnFormattedTimeStamp() + "done..." + newParagraphChar)

    return True


def moveToSyringeXYPosition(syringePosition):
    """

    :param syringePosition:
    :return:
    """
    print(returnFormattedTimeStamp() + "move to syringePosition X / Y" +
newLineChar)
    myReactoBot.write(
        bytes("G1 X" + str(syringePosition[0]) + " Y" + str(syringePosition[1])
+ " F10000\n", encoding="UTF-8"))
    # wait for movement to finish
    print(returnFormattedTimeStamp() + "...waiting..." + newLineChar)
    waitForMovementToFinish(myReactoBot, True)
    print(returnFormattedTimeStamp() + "done..." + newParagraphChar)

    return True


def moveToSyringeFillPosition():

    """


    :return:
    """
    raiseZForXYMovement()
    # move X back home
    myReactoBot.write(bytes("G1 X0 F10000\n", encoding="UTF-8"))
    # wait for movement to finish
    waitForMovementToFinish(myReactoBot, True)

    # lower Z into beaker
    myReactoBot.write(bytes("G1 Z0 F100\n", encoding="UTF-8"))
    # wait for movement to finish
    waitForMovementToFinish(myReactoBot, True)
```

```python
    return True


def dispenseChemical(syringePump):
    """

    :param syringePump:
    :return:
    """
    print(returnFormattedTimeStamp() + "dispensing chemical (homing syringe)..."
+ newLineChar)
    syringePump.write(homeSyringeCommand())
    # wait for movement to finish
    print(returnFormattedTimeStamp() + "...waiting..." + newLineChar)
    waitForMovementToFinish(syringePump)
    print(returnFormattedTimeStamp() + "done..." + newParagraphChar)

    return True


def fillSyringe(syringePump, amountOfChemical, overDraw=stdOverDraw,
numberOfFillEmptyCycles=stdNumberOfFillEmptyCycles,
equilibrationTime=stdEquilibrationTime):
    # homing the syringe
    """

    :param syringePump:
    :param amountOfChemical:
    :param overDraw:
    :param numberOfFillEmptyCycles:
    :param equilibrationTime:
    :return:
    """
    print(returnFormattedTimeStamp() + "homing the syringe..." + newLineChar)
    syringePump.write(homeSyringeCommand())
    # wait for homing to finish
    waitForMovementToFinish(syringePump)

    # setting a minimum cycling liquid of 4 mL (to accommodate glass syringes,
which need to wet the plunger to seal ok)
    totalAmountForCycling = amountOfChemical + overDraw
    #if totalAmountForCycling < 4:
    #    totalAmountForCycling = 4

    # running the fill cycles
    finishedCycles = 0
    while finishedCycles < numberOfFillEmptyCycles:
        # increment the cycle number so it's displayed correctly
        finishedCycles += 1
        print(returnFormattedTimeStamp() + "fill cycle " + str(finishedCycles) +
newLineChar)
        print(returnFormattedTimeStamp() + "drawing in " +
str(totalAmountForCycling) + " mL" + newLineChar)
        # actually telling the syringe to move
        syringePump.write(setAbsoluteCommand(totalAmountForCycling))
```

```
        # waiting for movement
        waitForMovementToFinish(syringePump)
        print(returnFormattedTimeStamp() + "waiting " + str(equilibrationTime) +
" s to equilibrate" + newLineChar)
        # equilibrating
        time.sleep(equilibrationTime)
        print(returnFormattedTimeStamp() + "expelling all liquid again" +
newLineChar)
        # expelling liquid again
        syringePump.write(homeSyringeCommand())
        # waiting for movement
        waitForMovementToFinish(syringePump)

    # filling the desired amount of chemical
    print(returnFormattedTimeStamp() + "drawing in correct amount (" +
str(amountOfChemical) + " mL)" + newLineChar)
    syringePump.write(setAbsoluteCommand(amountOfChemical))
    waitForMovementToFinish(syringePump)

    # waiting for pressure equilibration
    print(returnFormattedTimeStamp() + "waiting " + str(equilibrationTime) + " s
to equilibrate" + newLineChar)
    time.sleep(equilibrationTime)

    return True


def fillAllSyringes(acknowledgeEveryFill=True):
    """

    :param acknowledgeEveryFill:
    :return:
    """
    if runInDebugLevel > debugLevelTriggerBreaks:
        acknowledgeEveryFill = True

    ###
    ### filling syringe 1 with isoButylBenzene
    ###
    print(returnFormattedTimeStamp() + "ReactoBot ready to fill syringe 1
(isoButylBenzene, COM" +
          str(isoButylBenzenePort + 1) + ", " + str(isoButylBenzeneAmount) + "
mL)? The pump is going to over draw by 1 mL "
                                                                          "and
expel all liquid again three times before drawing in the correct amount of
chemical." + newLineChar)
    if acknowledgeEveryFill:
        input("The cycle starts with homing the syringe! Ready? Press Enter to
continue...")

    print(returnFormattedTimeStamp() + "Beginning fill cycle..." + newLineChar)
    if not debugSkipStep("filling of isoButylBenzenePump"):
        fillSyringe(isoButylBenzenePump, isoButylBenzeneAmount)
    print(returnFormattedTimeStamp() + "...filling cycle for isoButylBenzene
done." + newParagraphChar)
```

```
    ###
    ### done filling syringe 1 with isoButylBenzene
    ###

    ###
    ### filling syringe 2 with propanoicAcid
    ###
    print(returnFormattedTimeStamp() + "ReactoBot ready to fill syringe 2
(propanoicAcid, COM" +
          str(propanoicAcidPort + 1) + ", " + str(propanoicAcidAmount) + " mL)?
The pump is going to over draw by 1 mL "
                                                                          "and
expel all liquid again three times before drawing in the correct amount of
chemical." + newLineChar)
    if acknowledgeEveryFill:
        input("The cycle starts with homing the syringe! Ready? Press Enter to
continue...")

    print(returnFormattedTimeStamp() + "Beginning fill cycle..." + newLineChar)
    if not debugSkipStep("filling of propanoicAcidPump"):
        fillSyringe(propanoicAcidPump, propanoicAcidAmount)
    print(returnFormattedTimeStamp() + "...filling cycle for propanoicAcid
done." + newParagraphChar)
    ###
    ### done filling syringe 2 with propanoicAcid
    ###

    ###
    ### filling syringe 3 with triFluoroMethaneSulfonicAcid
    ###
    print(returnFormattedTimeStamp() + "ReactoBot ready to fill syringe 3
(triFluoroMethaneSulfonicAcid, COM" +
          str(triFluoroMethaneSulfonicAcidPort + 1) + ", " +
str(triFluoroMethaneSulfonicAcidAmount) + " mL)? The pump "

"is going to over draw by 1 mL and expel all liquid again three times before
drawing in the correct amount "

"of chemical." + newLineChar)
    if acknowledgeEveryFill:
        input("The cycle starts with homing the syringe! Ready? Press Enter to
continue...")

    print(returnFormattedTimeStamp() + "Beginning fill cycle..." + newLineChar)
    if not debugSkipStep("filling of triFluoroMethaneSulfonicAcidPump"):
        fillSyringe(triFluoroMethaneSulfonicAcidPump,
triFluoroMethaneSulfonicAcidAmount, 1)
    print(returnFormattedTimeStamp() + "...filling cycle for
triFluoroMethaneSulfonicAcid done." + newParagraphChar)
    ###
    ### done filling syringe 3 with triFluoroMethaneSulfonicAcid
    ###

    ###
    ### filling syringe 4 with diAcetoxyIodoBenzene
```

```
    ###
    print(returnFormattedTimeStamp() + "ReactoBot ready to fill syringe 4 
(diAcetoxyIodoBenzene, COM" +
          str(diAcetoxyIodoBenzenePort + 1) + ", " + 
str(diAcetoxyIodoBenzeneAmount) + " mL)? The pump is going to "
          
"over draw by 1 mL and expel all liquid again three times before drawing in the 
correct amount of chemical."
          + newLineChar)
    if acknowledgeEveryFill:
        input("The cycle starts with homing the syringe! Ready? Press Enter to 
continue...")

    print(returnFormattedTimeStamp() + "Beginning fill cycle..." + newLineChar)
    if not debugSkipStep("filling of diAcetoxyIodoBenzenePump"):
        fillSyringe(diAcetoxyIodoBenzenePump, diAcetoxyIodoBenzeneAmount)
    print(returnFormattedTimeStamp() + "...filling cycle for 
diAcetoxyIodoBenzene done." + newParagraphChar)
    ###
    ### done filling syringe 4 with diAcetoxyIodoBenzene
    ###

    ###
    ### filling syringe 5 with workUp
    ###
    print(returnFormattedTimeStamp() + "ReactoBot ready to fill syringe 5 
(workUp, COM" +
          str(workUpPort + 1) + ", " + str(workUpAmount) + " mL)? The pump is 
going to over draw by 1 mL and expel all "
                                                           "liquid again three 
times before drawing in the correct amount of chemical." + newLineChar)
    if acknowledgeEveryFill:
        input("The cycle starts with homing the syringe! Ready? Press Enter to 
continue...")

    print(returnFormattedTimeStamp() + "Beginning fill cycle..." + newLineChar)
    if not debugSkipStep("filling of workUpPump"):
        fillSyringe(workUpPump, workUpAmount)
    print(returnFormattedTimeStamp() + "...filling cycle for workUp done." + 
newParagraphChar)
    ###
    ### done filling syringe 5 with workUp
    ###

    return True


def performReaction1():
    """
        •   Into printed vessel is deposited 4-iso butyl benzene (syringe 1) and 
propanoic acid (syringe 2).
        •   [Pause to allow evaporation of low b.p. solvent]
        •   Slow (drop wise) addition of tri fluoro methane sulfonic acid 
(syringe 3)
        •   [agitation - 18hr]
```

```
    :return:
    """
    ##
    ## FIRST CHEMICAL
    ##
    print(returnFormattedTimeStamp() + "----------------------------" +
newLineChar)
    print(returnFormattedTimeStamp() + "    step 1 - chemical 1" + newLineChar)
    print(returnFormattedTimeStamp() + "----------------------------" +
newParagraphChar)

    if not debugSkipStep("step 1 - chemical 1"):
        # move to first syringe X / Y
        moveToSyringeXYPosition(isoButylBenzenePosition)
        # move to first syringe Z
        lowerZForChemicalDispensing(isoButylBenzenePosition)

        # dispense chemical (simply home syringe, as they are pre-filled)
        dispenseChemical(isoButylBenzenePump)

        # lift syringe up again
        raiseZForXYMovement()

    ##
    ## SECOND CHEMICAL
    ##
    print(returnFormattedTimeStamp() + "----------------------------" +
newLineChar)
    print(returnFormattedTimeStamp() + "    step 1 - chemical 2" + newLineChar)
    print(returnFormattedTimeStamp() + "----------------------------" +
newParagraphChar)

    if not debugSkipStep("step 1 - chemical 2"):
        # move to second syringe X / Y
        moveToSyringeXYPosition(propanoicAcidPosition)
        # move to second syringe Z
        lowerZForChemicalDispensing(propanoicAcidPosition)

        # dispense chemical (simply home syringe, as they are pre-filled)
        dispenseChemical(propanoicAcidPump)

        # lift syringe up again
        raiseZForXYMovement()


    if not debugSkipStep("wait for ether to evaporate"):
        # wait 60 minutes for di-ethyl-ether to evaporate
        evaporationTime = 3600
        agitateSample(myReactoBot, agitationTime=evaporationTime,
agitationAmplitude=15, agitationSpeed=3000)
        print(returnFormattedTimeStamp() + "evaporation time over." +
newLineChar)
```

```
    ##
    ## THIRD CHEMICAL
    ##
    print(returnFormattedTimeStamp() + "----------------------------" +
newLineChar)
    print(returnFormattedTimeStamp() + "    step 1 - chemical 3" + newLineChar)
    print(returnFormattedTimeStamp() + "----------------------------" +
newParagraphChar)

    if not debugSkipStep("step 1 - chemical 3"):
        # move to third syringe X / Y
        moveToSyringeXYPosition(triFluoroMethaneSulfonicAcidPosition)
        # move to third syringe Z
        lowerZForChemicalDispensing(triFluoroMethaneSulfonicAcidPosition)

        dispenseDropWiseQuery = ""

        while dispenseDropWiseQuery != "yes":
            dispenseDropWiseQuery = input("Do you want to dispense the chemical
3 for step 1 drop wise? (type \"yes\" or \"no\" and press enter)\n")

            if dispenseDropWiseQuery == "no":
                break

        if dispenseDropWiseQuery == "yes":
            print(returnFormattedTimeStamp() + "dispensing chemical drop wise."
+ newLineChar)
            # dispense chemical drop wise (over 10 minutes)
            dispenseDropWise(triFluoroMethaneSulfonicAcidPump,
triFluoroMethaneSulfonicAcidAmount, 10)
        else:
            dispenseChemical(triFluoroMethaneSulfonicAcidPump)
            print(returnFormattedTimeStamp() + "dispensing chemical directly." +
newLineChar)

        # lift syringe up again
        raiseZForXYMovement()

    # move X back home
    print(returnFormattedTimeStamp() + "Moving X-axis back home..." +
newLineChar)
    myReactoBot.write(bytes("G1 X0 F10000\n", encoding="UTF-8"))
    # wait for movement to finish
    print(returnFormattedTimeStamp() + "...waiting..." + newLineChar)
    waitForMovementToFinish(myReactoBot, True)
    print(returnFormattedTimeStamp() + "done..." + newLineChar)

    if not debugSkipStep("agitate sample for 18 h after step 1"):
        # shake sample for 18 h (= 64800 seconds)
        agitateSample(myReactoBot, agitationTime=64800, agitationAmplitude=15,
agitationSpeed=3000)

    return True
```

```
def performReaction2():
    """
        •   Slow (drop wise) addition of PhI(OAc)2
(diAcetoxyIodoBenzenePosition) in tri methyl ortho formate (syringe 4)
        •   [agitation - 3hr]


    :return:
    """

    ##
    ## FIRST CHEMICAL
    ##
    print(returnFormattedTimeStamp() + "----------------------------" +
newLineChar)
    print(returnFormattedTimeStamp() + "     step 2 - chemical 1" + newLineChar)
    print(returnFormattedTimeStamp() + "----------------------------" +
newParagraphChar)

    if not debugSkipStep("step 2 - chemical 1"):
        # move to first syringe X / Y
        moveToSyringeXYPosition(diAcetoxyIodoBenzenePosition)
        # move to first syringe Z
        lowerZForChemicalDispensing(diAcetoxyIodoBenzenePosition)

        dispenseDropWiseQuery = ""

        while dispenseDropWiseQuery != "yes":
            dispenseDropWiseQuery = input("Do you want to dispense the chemical
for step 2 drop wise? (type \"yes\" or \"no\" and press enter)\n")

            if dispenseDropWiseQuery == "no":
                break

        if dispenseDropWiseQuery == "yes":
            print(returnFormattedTimeStamp() + "dispensing chemical drop wise."
+ newLineChar)
            # dispense chemical drop wise (over 4 minutes)
            dispenseDropWise(diAcetoxyIodoBenzenePump,
diAcetoxyIodoBenzeneAmount, 10)
        else:
            dispenseChemical(diAcetoxyIodoBenzenePump)
            print(returnFormattedTimeStamp() + "dispensing chemical directly." +
newLineChar)

        # lift syringe up again
        raiseZForXYMovement()

    # move X back home
    myReactoBot.write(bytes("G1 X0 F10000\n", encoding="UTF-8"))
    # wait for movement to finish
    waitForMovementToFinish(myReactoBot, True)

    if not debugSkipStep("agitate sample for 3 h after step 2"):
```

```
        # shake sample for 3 h (= 10800 seconds)
        agitateSample(myReactoBot, agitationTime=10800, agitationAmplitude=15,
agitationSpeed=3000)

    return True


def performReaction3():
    """
        •   Addition of KOH in MeOH/H2O (syringe 5)


    :return:
    """
    ##
    ## FIRST CHEMICAL
    ##
    print(returnFormattedTimeStamp() + "----------------------------" +
newLineChar)
    print(returnFormattedTimeStamp() + "     step 3 - chemical 1" + newLineChar)
    print(returnFormattedTimeStamp() + "----------------------------" +
newParagraphChar)

    if not debugSkipStep("step 3 - chemical 1"):
        # move to first syringe X / Y
        moveToSyringeXYPosition(workUpPosition)
        # move to first syringe Z
        lowerZForChemicalDispensing(workUpPosition)

        # dispense chemical (simply home syringe, as they are pre-filled)
        dispenseChemical(workUpPump)

        # lift syringe up again
        raiseZForXYMovement()

    # move X back home
    myReactoBot.write(bytes("G1 X0 F10000\n", encoding="UTF-8"))
    # wait for movement to finish
    waitForMovementToFinish(myReactoBot, True)


    if not debugSkipStep("agitate samples for 1 h after step 3"):
        # shake sample for 1 h (= 3600 seconds)
        agitateSample(myReactoBot, agitationTime=3600, agitationAmplitude=15,
agitationSpeed=3000)


    return True


if __name__ == '__main__':
    ###
    # sending gCode and starting the print
    ###
    # this assumes that OctoPrint is already running and connected
```

```
    '''
    if not debugSkipStep("printing of beaker"):
        print(returnFormattedTimeStamp() + "connecting to OctoPrint..." +
newLineChar)
        myOctoPrintClient = OctoClient(host=octoPrintAddress,
port=octoPrintPort, apiKey=octoPrintAPIKey)
        print(returnFormattedTimeStamp() + "uploading gCode to OctoPrint..." +
newLineChar)
        myOctoPrintClient.upload(pathName=gCodeFilePath,
octoPrintFileName=gCodeFileName, startPrint=True)
        print(returnFormattedTimeStamp() + "printing..." + newLineChar)

        # inform the user of the print progress
        previousCompletion = 0
        while myOctoPrintClient.status()['progress']['completion'] < 100.0:
            if myOctoPrintClient.status()['progress']['completion'] ==
previousCompletion:
                time.sleep(10)
            else:
                previousCompletion =
myOctoPrintClient.status()['progress']['completion']
                print(returnFormattedTimeStamp() +
myOctoPrintClient.status()['progress']['completion'] + " %..." + newLineChar)


        # inform the user that the print is done
        print(newLineChar + returnFormattedTimeStamp() + "print done
disconnecting from OctoPrint..." + newLineChar)
        # tell OctoPrint to disconnect from the printer so the printers' serial
port unblocks
        myOctoPrintClient.disconnect()
        print(returnFormattedTimeStamp() + "disconnected..." + newParagraphChar)
    '''
    # needs to be created after OctoPrint is done, since the port is blocked
while printing
    print(returnFormattedTimeStamp() + "connecting directly to ReactoBot..." +
newLineChar)
    myReactoBot = serial.Serial(reactoBotPort, 115200)
    print(returnFormattedTimeStamp() + "...waiting..." + newLineChar)
    time.sleep(2)
    print(returnFormattedTimeStamp() + "connected" + newParagraphChar)

    if not debugSkipStep("initial homing of all axis"):
        # homing the printer to recover from a possible loss of position
        print(returnFormattedTimeStamp() + "homing X, Y, Z..." + newLineChar)
        myReactoBot.write(b"G28 F10000\n")
        # wait for movement to finish
        print(returnFormattedTimeStamp() + "...waiting..." + newLineChar)
        waitForMovementToFinish(myReactoBot, True)
        print(returnFormattedTimeStamp() + "done" + newParagraphChar)

    # filling the syringes for the synthesis
```

```
    print(returnFormattedTimeStamp() + "###############" + newLineChar)
    print(returnFormattedTimeStamp() + "###############" + newLineChar)
    print(returnFormattedTimeStamp() + "#### preparation... filling all syringes
with the needed chemicals" + newParagraphChar)
    print(returnFormattedTimeStamp() + "###############" + newLineChar)
    if not debugSkipStep("fill all syringes"):
        fillAllSyringes(acknowledgeEveryFill=True)
    print(returnFormattedTimeStamp() + "###############" + newLineChar)
    print(returnFormattedTimeStamp() + "#### preparation done. all syringes
filled" + newLineChar)
    print(returnFormattedTimeStamp() + "###############" + newLineChar)
    print(returnFormattedTimeStamp() + "###############" + newParagraphChar)

    if not debugSkipStep("raising Z to clear print"):
        # raising Z to clear print
        raiseZForXYMovement()

    if not debugSkipStep("centering over print"):
        print(returnFormattedTimeStamp() + "centering X and Y over print
(assuming print was made in the center of the build plate!)..." + newLineChar)
        myReactoBot.write(b"G1 X100 Y100 F10000\n")
        # wait for movement to finish
        print(returnFormattedTimeStamp() + "...waiting..." + newLineChar)
        waitForMovementToFinish(myReactoBot, True)
        print(returnFormattedTimeStamp() + "done" + newParagraphChar)

    ###
    # reaction step 1
    ###
    print(returnFormattedTimeStamp() + "########" + newLineChar)
    print(returnFormattedTimeStamp() + "########" + newLineChar)
    print(returnFormattedTimeStamp() + "#### starting reaction step 1..." +
newParagraphChar)
    if not debugSkipStep("perform reaction step 1"):
        performReaction1()
    print(returnFormattedTimeStamp() + "#### reaction step 1 done" +
newLineChar)
    print(returnFormattedTimeStamp() + "########" + newLineChar)
    print(returnFormattedTimeStamp() + "########" + newParagraphChar)

    ###
    # reaction step 2
    ###
    print(returnFormattedTimeStamp() + "########" + newLineChar)
    print(returnFormattedTimeStamp() + "########" + newLineChar)
    print(returnFormattedTimeStamp() + "#### starting reaction step 2..." +
newParagraphChar)
    if not debugSkipStep("perform reaction step 2"):
        performReaction2()
    print(returnFormattedTimeStamp() + "#### reaction step 2 done" +
newLineChar)
    print(returnFormattedTimeStamp() + "########" + newLineChar)
    print(returnFormattedTimeStamp() + "########" + newParagraphChar)

    ###
```

```
    # reaction step 3
    ###
    print(returnFormattedTimeStamp() + "########" + newLineChar)
    print(returnFormattedTimeStamp() + "########" + newLineChar)
    print(returnFormattedTimeStamp() + "#### starting reaction step 3..." +
newParagraphChar)
    if not debugSkipStep("perform reaction step 3"):
        performReaction3()
    print(returnFormattedTimeStamp() + "#### reaction step 3 done" +
newLineChar)
    print(returnFormattedTimeStamp() + "########" + newLineChar)
    print(returnFormattedTimeStamp() + "########" + newParagraphChar)

    print(returnFormattedTimeStamp() + "################################" +
newLineChar)
    print(returnFormattedTimeStamp() + "################################" +
newLineChar)
    print(returnFormattedTimeStamp() + "    ibuprofen synthesis done" +
newLineChar)
    print(returnFormattedTimeStamp() + "################################" +
newLineChar)
    print(returnFormattedTimeStamp() + "################################" +
newParagraphChar)

    # release motors
    print(returnFormattedTimeStamp() + "releasing all motors" + newLineChar)
    myReactoBot.write(b"M84\n")
    print(returnFormattedTimeStamp() + "...waiting..." + newLineChar)
    waitForMovementToFinish(myReactoBot)
    print(returnFormattedTimeStamp() + "done" + newParagraphChar)
```

Design software

The 3D-printed reactors used in this work were designed using the FreeCAD software package although any 3D modelling/CAD software with the ability to export models in an .STL file format would suffice for this, and there are a number of other suitable alternative free/open source candidates available on the internet. Reactor designs were translated into 3D printer instruction files (G-code) using Slic3r software (available free from http://slic3r.org/).

Device model

Three similar reaction vessel 3D models were designed to be printed on the robotic platform of different total internal capacities. The vessels were designed such that their external dimensions were similar, but contained differing sized cavities within. Once printed the reaction vessels conformed to with 5% of the dimensions of the 3D model designs. A fourth reactor vessel with larger external and internal capacities was also produced in order to further scale up the reaction, however it was found that the use of this reactor decreased the yield obtained (see below).

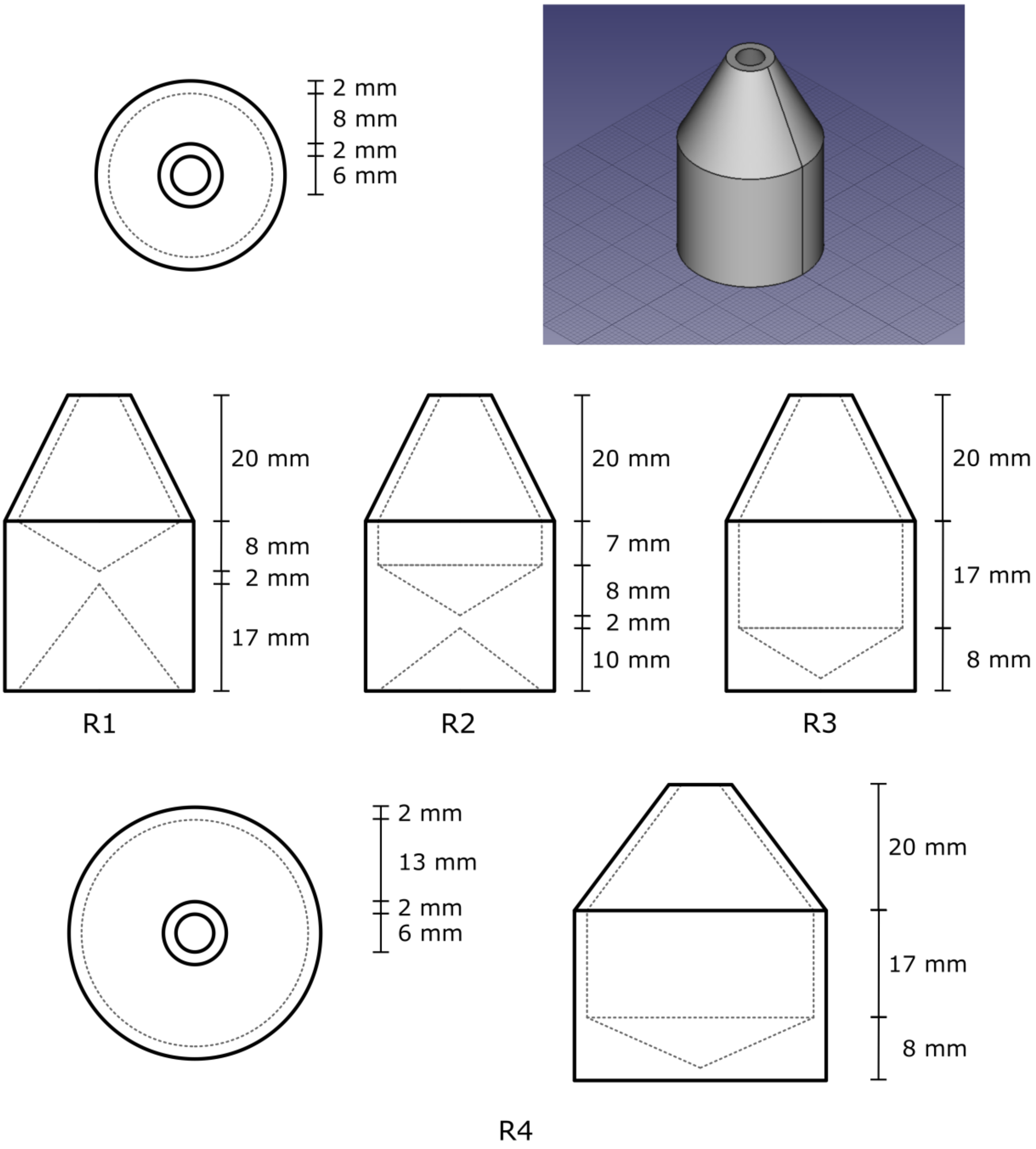

Figure S4:*Top left: Top view Annotated CAD design of the reaction vessels. Top right: render of the exterior of the 3D printed reaction vessels. Bottom: Side view annotated CAD design of the 3D printed reaction vessels R1, R2 and R3 and Top and side view of reaction vessel R4*

Print settings

The device was printed in polypropylene (PP, supplied by Barnes Plastic Welding Equipment Ltd., Blackburn , UK), extruded at 260 °C onto a 12 mm thick PP print bed. A selection of significant settings for PP printing on a RepRap type 3D printer are given below:

Layer Height:	0.2 mm
First Layer Height:	0.35 mm
Perimeters:	5
Fill Density:	100%
Perimeter Speed:	60 mm/s
Small Perimeter Speed:	60 mm/s
External Perimeter Speed:	70%
Infill Speed:	120 mm/s
Travel Speed:	130 mm/s
First Layer Speed:	30%

Other printing parameters were either redundant or had no significant impact on the print quality of the objects printed

Ibuprofen synthesis

The synthesis of ibuprofen in the automated robotic platform was achieved by running the control software as described above. Once the reaction vessel was printed the syringe pumps were filled with the relevant solutions by placing the solutions in vials into which the appropriate dispensing syringe was inserted to allow the uptake of the appropriate volume of material. Care was required when filling the trifluoromethanesulfonic acid syringe pump. The trifluoromethanesulfonic acid was first transferred into a vial which had been purged with dry nitrogen and fitted with a septum and a balloon filled with dry nitrogen to minimize contact with atmospheric moisture. The dispensing syringe was then inserted into the septum to withdraw the required material. Residual atmospheric moisture in the dead volume of the tubing tended to cause some fuming within the vial as the trifluoromethansulfonic acid was withdrawn, however this was minimal and contained within the vial. Once the syringe pumps were loaded with the solutions the control software continued with the synthesis as described in the manuscript.

Initial test syntheses were performed using the debug mode described for the process control software where each stage of the program could be skipped individually in order to isolate the particular reaction stages. To this end the reaction mixtures at the end of individual stages were analysed by ^{1}H NMR in MeOD-d_4 to confirm the reaction progress. All test reactions were performed in the 3D printed reaction ware as part of the automated sequence.

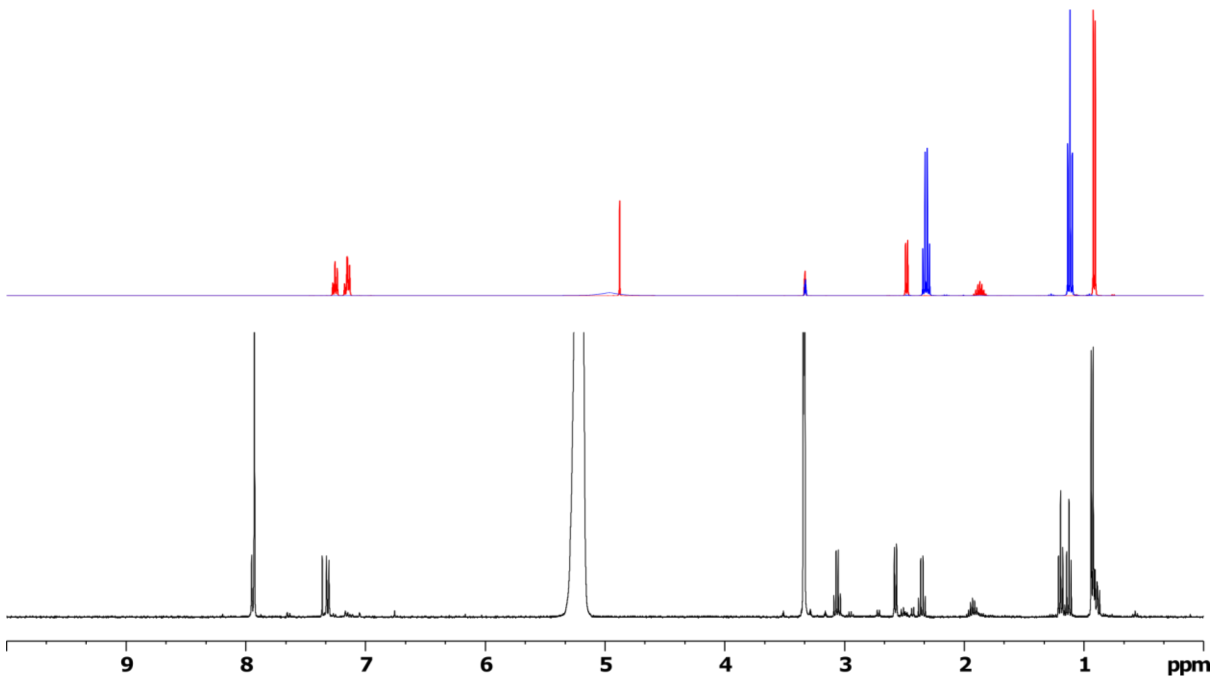

***Figure S5**:^{1}H* NMR (400 MHz, MeOD *d4*) comparison of friedel-crafts acylation of isobutyl benzene with propionic acid. Top: reference spectra of isobutylbenzene (red spectrum) and propionic acid (blue spectrum). Bottom: crude reaction mixture after completion of 1st stage of the synthesis procedure as performed in PP reaction vessel under automated conditions. Yield by NMR for this reaction step is calculated to be approximately 71 %

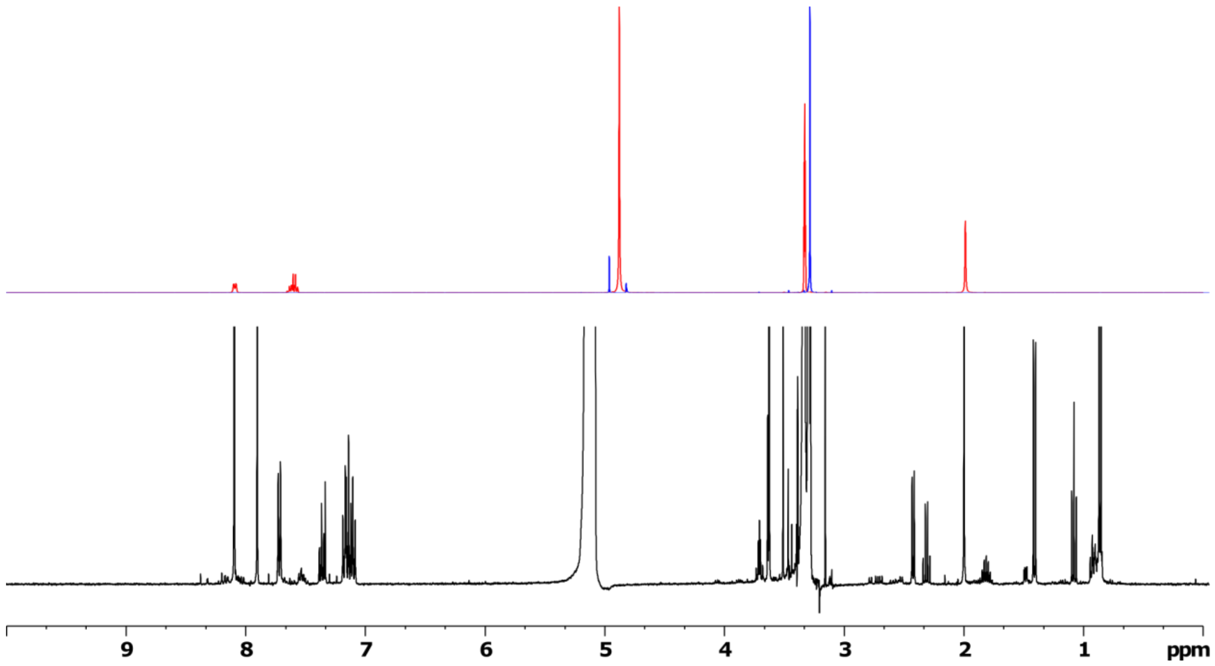

Figure S6: ^{1}H NMR (400 MHz, MeOD d_4) comparison of 1,2-aryl migration of the product of step 1 with the reagents $PhI(OAc)_2$ and trimethyl orthoformate.. Top: reference spectra of trimethyl orthoformate (red spectrum) and $PhI(OAc)_2$ (blue spectrum). Bottom: crude reaction mixture after completion of 2nd stage of the synthesis procedure as performed in PP reaction vessel under automated conditions. NMR yield for this reaction was calculated to be approximately 64%

After completion of the automated reaction process, the crude reaction mixture was retrieved from the reaction vessel by pipette and MeOH was removed in vacuo. To the solution was added water and the aqueous layer was washed with Et_2O and made acidic by the addition of concentrated HCl. The aqueous layer was then extracted with Et_2O. The combined ethereal layers were washed with distilled H_2O and brine, dried over $MgSO_4$, filtered and washed with Et_2O, and concentrated in vacuo. The residue was purified by reversed phase column chromatography on C_{18} (60% MeCN/H_2O) to give acid **4** (^{1}H and ^{13}C NMR spectra of retrieved ibuprofen are shown in Figure S7 and S8). The automated procedures on the three different volume scales were repeated 6 times each. An average yield could not be obtained for reactions with larger volume reaction vessels, as these reactors did not give reproducible yields.

((*R,S*)-2-(4-(2-Methylpropyl)phenyl)propanoic acid), ibuprofen (4); ^{1}H NMR ($CDCl_3$, 400 MHz): δ 7.24(d, 2H, *J* = 4.6 Hz) 7.13 (d, 2H, *J*=8.1 Hz), 3.74 (m, 4H), 2.47 (d, 2H, *J*=7.2 Hz), 1.87 (m, 1H), 1.53 (d, 3H, *J*=7.2 Hz), 0.92 (d, 3H, *J*= 6.6); **^{13}C NMR ($CDCl_3$, 100 MHz):** δ 180.4, 140.9, 137.0, 129.4, 127.3, 45.0, 44.9, 30.2, 22.4, 18.1; **HMRS (MH+) calcd. for $C_{13}H_{19}O_2$:** 207.1385, **found:** 207.1640. **Isolated yield:** See Table 3 in the manuscript.

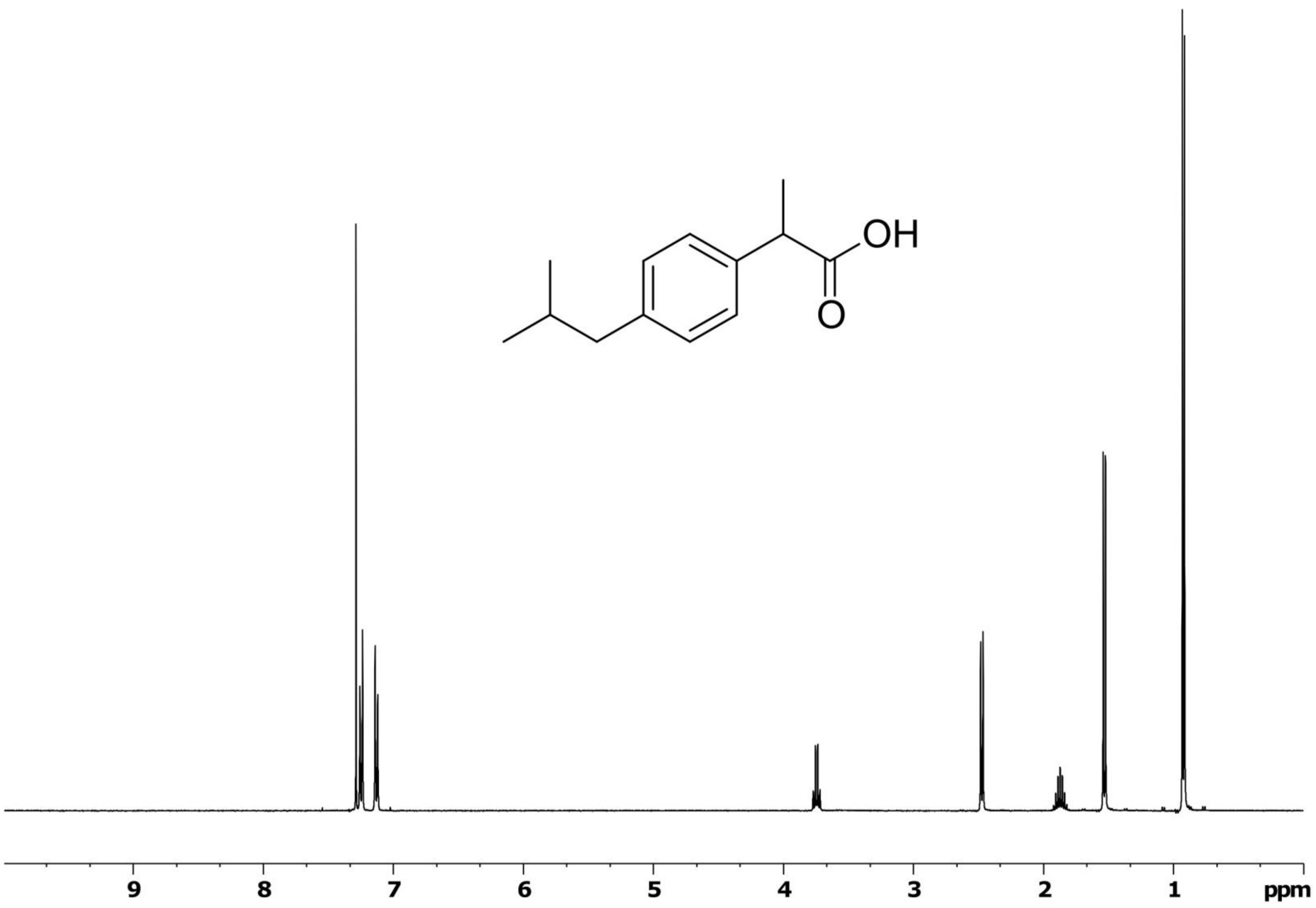

Figure S7: ^1H NMR (400 MHz, $CDCl_3$) of a purified ibuprofen sample obtained from automated synthesis robot.

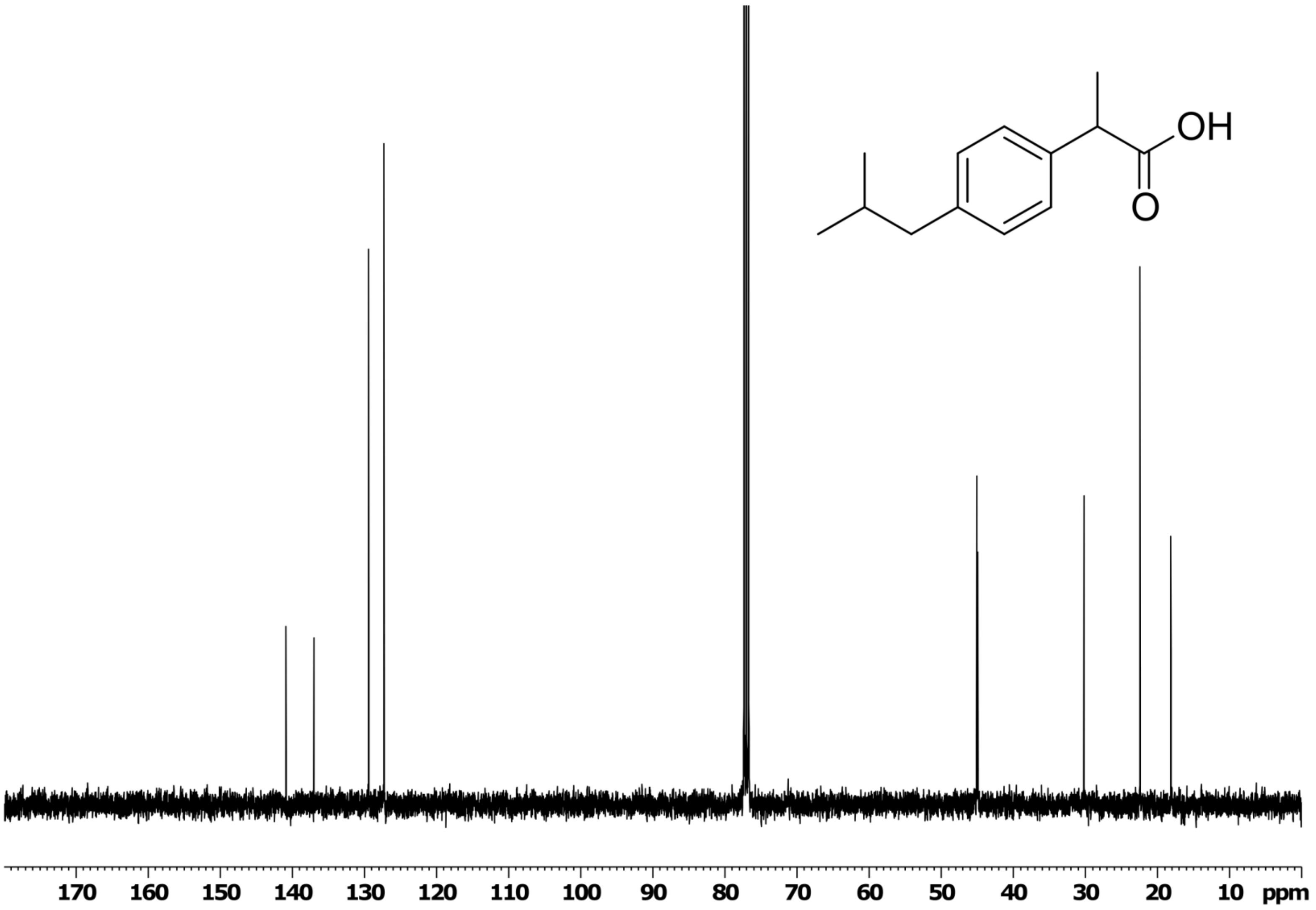

Figure S8: ^{13}C NMR (400 MHz, $CDCl_3$) of a purified ibuprofen sample obtained from automated synthesis robot.
